# P-846. Impact of an Ambulatory Antimicrobial Stewardship Intervention on Respiratory Antibiotic Prescribing in Primary Care Clinics

**DOI:** 10.1093/ofid/ofaf695.1054

**Published:** 2026-01-11

**Authors:** Hana R Winders, Pamela Bailey, Pamela Bailey, Joseph Kohn, Sean J Battle, Carmen M Faulkner-Fennell, Jamee Steen, Gregg M Talente, Majdi N Al-Hasan

**Affiliations:** Prisma Health Midlands, Columbia, SC; University of South Carolina School of Medicine, Columbia, South Carolina; University of South Carolina School of Medicine, Columbia, South Carolina; Prisma Health Midlands, Columbia, SC; Prisma Health/University of South Carolina, Columbia, South Carolina; Prisma Health-Upstate, Greer, South Carolina; Prisma Health/University of South Carolina School of Medicine, Columbia, South Carolina; University of South Carolina School of Medicine, Columbia, South Carolina; University of South Carolina School of Medicine, Columbia, South Carolina

## Abstract

**Background:**

Inappropriate antibiotic prescribing is common in ambulatory settings, most often with respiratory tract diagnoses (RTDs). The goal of this study was to assess the impact of an antimicrobial stewardship intervention on prescribing for RTDs in primary care clinics.

**Figure:**
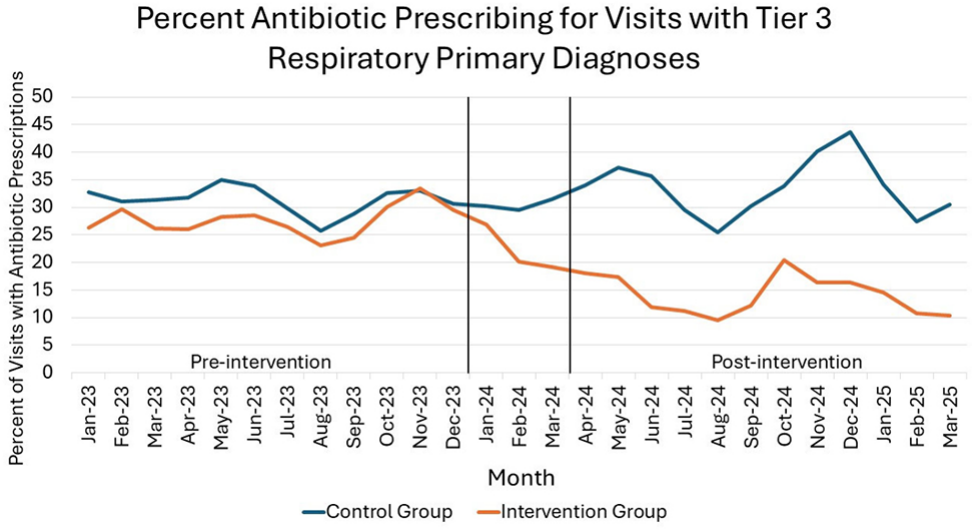

**Methods:**

This was a quasi-experimental, pre/post, retrospective cohort study from January 2023 - March 2025 with an implementation period from January - March 2024. RTDs were grouped into tier 1 (antibiotics always indicated), tier 2 (maybe indicated) and tier 3 (never indicated). Twenty clinics received a multifaceted intervention including monthly provider feedback reports on antibiotic prescribing for tier 3 RTDs, and 54 clinics were in the control group. The primary outcome was the rate of antibiotic prescribing for tier 3 RTDs. Secondary outcomes include antibiotic prescribing for all, tier 1 and tier 2 RTDs. χ2 test and matched pairs mean differences were used to compare the rate of visits with antibiotic prescriptions overall and for each clinic, respectively, before and after the intervention.

**Results:**

Of patients with tier 3 RTDs, the median age was 55 in the intervention group and 50 in the control group with 71.1% and 62.7% female, respectively. Antibiotic prescribing in visits with tier 3 RTDs as primary diagnoses decreased in the intervention group (3544/12677 [28%] vs 1770/12599 [14%]; p< 0.001) and slightly increased in the control group (16973/54023 [31.4%] vs 16913/50584 [33.4%]; p< 0.001; Figure). Excluding visits with tier 1 or 2 secondary RTDs showed a similar result (intervention: 24.6% vs 10%, p< 0.001; control: 29% vs 30.9%, p< 0.001). Comparable results were seen when analyzed using matched pairs mean difference (intervention: -11.7%, 95% CI: -15.8 to -7.7%, p< 0.001; control: 1.8%, 95% CI: 0.1 to 3.4%, p= 0.04). Antibiotic prescribing for all RTDs decreased from 41.7% to 33.2% in the intervention group while increasing in the control group from 47.5% to 48.9% (p< 0.001). A decrease was also seen in the intervention group for tier 2 RTDs along with a slight increase in the control group (p< 0.001); however, the intervention group did not see a change for tier 1 RTDs (p=0.54).

**Conclusion:**

A multifaceted antimicrobial stewardship intervention was associated with a 50% decline in antibiotic prescribing for tier 3 RTDs.

**Disclosures:**

All Authors: No reported disclosures

